# Association of cord blood Ang-1 and sCD105 levels with bronchopulmonary dysplasia in preterm infants

**DOI:** 10.1186/s12887-024-04932-7

**Published:** 2024-07-15

**Authors:** Jingyun Yang, Yun Wang, Yixin Wu, Hailing Fan, Ouxuan Jin, Liwei Tang, Tao-Hsin Tung, Meixian Zhang, Lizhen Wang

**Affiliations:** 1 Department of Pediatrics, Taizhou Hospital of Zhejiang Province Affiliated to Wenzhou Medical University, Linhai, Zhejiang 317000 China; 2 Evidence-Based Medicine Center, Taizhou Hospital of Zhejiang Province Affiliated to Wenzhou Medical University, 150 Ximen Street, Linhai, Zhejiang 317000 China; 3 Department of Pediatrics, Taizhou Enze Medical Center (Group), Enze Hospital, Taizhou Hospital of Zhejiang Province affiliated to Wenzhou Medical University, Taizhou, Zhejiang 318050 China

**Keywords:** Preterm infants, Bronchopulmonary dysplasia, Ang-1, sCD105, Early prediction

## Abstract

**Background:**

To investigate the relationship between cord blood levels of Angiopoietin-1 (Ang-1) and S-endoglin (sCD105) and bronchopulmonary dysplasia (BPD) in preterm infants.

**Methods:**

Sixty-one preterm infants admitted to the neonatal intensive care unit of the study hospital between July 2021 and September 2022 were included. Cord blood was collected after the birth of premature infants. Ang-1 and sCD105 levels were quantified using the vascular endothelial growth factor enzyme-linked immunosorbent assay. Preterm infants were divided into BPD and non-BPD groups, and differences in Ang-1 and sCD105 levels between the two groups were compared. A binary logistic model was used to assess the association between low and high levels Ang-1 and BPD in preterm infants.

**Results:**

In the study, there were 20 preterm infants with BPD (32.8%) and 41 preterm infants with non-BPD (67.2%). Ang-1 concentration levels were lower in the BPD group than in the non-BPD group (7105.43 (5617.01–8523.00) pg/ml vs. 10488.03 (7946.19–15962.77) pg/ml, *P* = 0.027). However, the sCD105 concentration levels were not significantly different between the BPD and non-BPD groups (*P* = 0.246). A median Ang-1 concentration of 8800.40 pg/ml was calculated. Logistic regression analysis showed that after adjusting for gestational age, birth weight, and maternal prenatal steroid hormone application, the odds ratio (OR) was 8.577 for the risk of BPD in preterm infants with Ang-1 concentrations of ≤ 8800.40 pg/ml compared to those with Ang-1 concentrations of > 8800.40 pg/ml (OR: 8.577, 95% confidence interval: 1.265–58.155, *P* = 0.028).

**Conclusion:**

Our study indicated that Ang-1 levels in the cord blood of preterm infants may be associated the risk of BPD. In the future, we will continue to conduct study with large samples.

## Introduction

Bronchopulmonary dysplasia (BPD) remains one of the most serious pulmonary complications in preterm infants, with extremely high morbidity and mortality [1], and can lead to infants developmental delay and death, especially in very early preterm infants. However, as the survival rate of preterm infants increases, the incidence of BPD also rises significantly [2]. Our multicenter survey in 2019 reported a BPD prevalence as high as 72.2% in ultra-immature or ultra-low birth weight infants [3]. A large U.S. study reported an incidence rate of 55% for BPD in preterm infants born at 26 weeks of gestation [4]. There have been great advances in the study of BPD, and the emergence of prenatal steroid hormones, Continuous Positive Airway Pneumatic Therapy, and replacement therapy with surfactant substances have all led to breakthroughs in BPD treatment [5]. BPD is the leading cause of prolonged hospitalization, growth retardation, recurrent respiratory illnesses, and neonatal mortality in children [6]. If biomarkers for the early prediction of BPD can be sought and high-risk preterm infants who may progress to BPD can be screened for early prediction, early intervention, and early treatment, the incidence of BPD can be reduced to a certain extent. This would reduce the severity of the disease and improve the long-term prognosis of children with BPD.

The pathogenesis of BPD remains unclear, and studies related to the mechanism of BPD at home and abroad have mainly focused on inflammation, pulmonary fibrosis, angiogenesis, and alveolar development-related factors [7]. BPD was conceptualized in 2001, and its pathogenesis involves the abnormal development of alveoli and pulmonary microvasculature, resulting in alveolar hypoplasia and vascular dysfunction [8]. Much current research has focused on impaired angiogenesis and the link between BPD and pulmonary vascular disease [9]. An inflammation-induced imbalance between pro- and antiangiogenic factors is a potential mechanism for impaired angiogenesis [10].

Angiopoietin (Ang), a class of paracrine growth factors, are mainly secreted by vascular smooth muscle cells and other vascular endothelial cells, and act on endothelial cells [11]. Ang-1 is a cytokine with strong provascular growth effects. It has been found that Ang-1 promotes the differentiation of vascular smooth muscle cells and mesenchymal stromal cells, which have important roles in angiogenesis, development, maturation, and maintenance of the integrity of the vascular system [12].

S-endoglin (sCD105) is an anti-angiogenic molecule involved in the regulation of angiogenesis and neovascularization, and is highly expressed on the cell membrane of vascular endothelial and syncytiotrophoblast cells. The expression of sCD105 has been found to be elevated in several diseases with vascular involvement, such as type 1 diabetic nephropathy, preeclampsia, and patients with coronary atherosclerosis [13, 14].

Therefore, the present study hypothesized that the expression levels of Ang-1 and sCD105 in the umbilical cord blood might be associated with the development of BPD. Currently, the expression levels of umbilical cord blood Ang-1 and sCD105 in preterm infants with BPD have rarely been reported. In this study, we selected preterm infants admitted to the neonatal intensive care unit of Taizhou Hospital to investigate the correlation between Ang-1 and sCD105 levels in umbilical cord blood and BPD in preterm infants to provide early clues for the prediction of BPD.

## Materials and methods

### Subjects and data collection

From July 2021 to September 2022, preterm infants (≤ 34 weeks gestation) who were delivered in the department of obstetrics and gynecology of Taizhou Hospital in the Zhejiang Province and were transferred to the neonatal intensive care unit within 24 h were selected for the study. This study was approved by the Ethics Committee of Taizhou Hospital (Ethics filing approval no. K20201204), and written informed consent was obtained from all parents/guardians of all the study participants.

We collected clinical data of preterm infants and their mothers using the hospital’s electronic medical record system. (1) Clinical data of preterm infants included the gestational age (< 30, 30–32, and 32–34 weeks), birth weight (< 1000, 1000–1500, and >1500 g), sex, Apgar score at 1 min (≤ 7, 8–10), and days of hospitalization. (2) Clinical data of mothers included abortions, delivery method (cesarean section and natural birth), premature rupture of membranes, antenatal steroids, maternal diabetes, and maternal hypertension.

### Diagnosis of BPD and grouping

According to the diagnostic criteria for BPD [15], any preterm infant that has oxygen dependence (an inhaled oxygen concentration of > 21%) for > 28 days. The enrolled participants were categorized into two groups: BPD and non-BPD.

### Exclusion criteria

(1) inherited metabolic diseases and chromosomal abnormalities; (2) respiratory malformations; (3) other lethal congenital malformations (e.g., central nervous system malformations); (4) prolonged use of oxygen due to non-pulmonary disorders, such as central apnea, diaphragmatic paralysis, and other conditions; and (5) incomplete clinical data.

### Experimental methods

Exactly 2 ml of umbilical cord blood was collected from preterm infants at birth, it was then anticoagulated using EDTA, centrifuged horizontally at 1500 rpm/min for 15 min at 4 °C, and the plasma was aspirated and frozen in a -80 °C storage cabinet. An enzyme-linked immunosorbent assay was used to detect the concentration levels of Ang-1 (Abcam, UK) and sCD105 (Abcam, UK), and the results were expressed in pg/ml.

### Statistical analysis

Continuous variables that conformed to normal distribution were expressed as mean ± standard deviation (SD); the t-test was used to evaluate the difference between the BPD and non-BPD groups, and those that did not conform to normal distribution were expressed using the median (interquartile spacing), and the rank-sum test was used to evaluate the difference between the two groups. Categorical variables are expressed as counts and percentages. The Chi-square test was used to assess differences between the two groups. Ang-1 and sCD105 concentrations were log-transformed to ensure normal distribution. Logistic regression analysis was used to evaluate the association between low concentration levels of Ang-1 and the risk of developing BPD in preterm infants, and 95% adjusted odds ratios (OR) and confidence intervals (CI) were calculated. The following model sequences were constructed: Model 1 was a crude model, Model 2 was adjusted for the gestational age and birth weight of preterm infants, and Model 3 was additionally corrected for antenatal steroid application by the mother. All data were statistically analyzed using IBM SPSS software (version 26.0, SPSS Inc.). Statistical significance was set at *P* < 0.05.

## Results

### General basic information

**Fig. 1:**
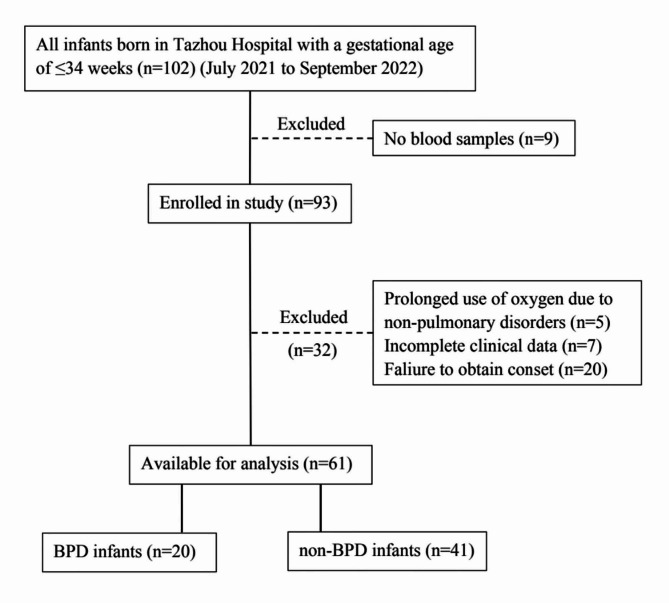
Flow diagram showing the study design involving the 61 infants screened in this study

A total of 61 preterm infants were included in this study (Fig. 1). Table 1 summarizes the basic data of the preterm infants in the BPD and non-BPD groups. In this study, 20 preterm infants had BPD (32.8%) and 41 preterm infants did not (67.2%). There were significant differences in gestational age (30.94 ± 1.36 weeks vs. 32.90 ± 0.90 weeks) and birth weight (1517.25 ± 407.19 g vs. 1968.05 ± 391.88 g) between the preterm infants in the BPD and non-BPD groups, respectively (*P* < 0.001). There was no statistical difference between the two groups in terms of the sex of preterm infants, 1-minute Apgar score, mode of delivery, history of previous miscarriages and premature rupture of membranes in the mothers.

**Table 1:** Comparison of general information on preterm infants in the BPD and non-BPD groups (*n* = 61)

Variables		BPD group (*n* = 20)		Non-BPD group (*n* = 41)		*P*-value		
**Preterm infants characteristics**								
Gestational age (weeks)		30.94 ± 1.36		32.90 ± 0.90		**< 0.001**		
Birth weight (g)		1517.25 ± 407.19		1968.05 ± 391.88		**< 0.001**		
Sex						0.628		
Boy		13(65.0)		24(58.5)				
Girl		7(35.0)		17(41.5)				
Gestational age						**< 0.001**		
< 30 weeks		5(25.0)		0(0.0)				
30–32 weeks		10(50.0)		7(17.1)				
32–34 weeks		5(25.0)		34(82.9)				
Birth weight (g)						**0.001**		
< 1000		2(10.0)		0(0.0)				
1000–1500		9(45.0)		5(12.2)				
>1500		9(45.0)		36(87.8)				
Apgar score at 1 min						0.720		
≤ 7		2(10.0)		3(7.3)				
8–10		18(90.0)		38(92.7)				
Length of hospital stay (days)		39.5(32.25–54.75)		22(17–27)		**< 0.001**		
**Maternal characteristics**								
Abortions						0.921		
Yes		11(55.0)		22(53.7)				
No		9(45.0)		19(46.3)				
Delivery method						0.090		
Cesarean section		14(70.0)		36(87.8)				
Natural birth		6(30.0)		5(12.2)				
Premature rupture of membranes						0.402		
Yes		8(40.0)		12(29.3)				
No		12(60.0)		29(70.7)				
Antenatal steroids						**0.018**		
Yes		11(55.0)		10(24.4)				
No		9(45.0)		31(75.6)				
Maternal diabetes						0.640		
Yes		6(30.0)		10(24.4)				
No		14(70.0)		31(75.6)				
Maternal hypertension						0.302		
Yes		3(15.0)		11(26.8)				
No		17(85.0)		30(73.2)				
**Preterm complications**								
NRDS		19(95.0)		35(85.4)		0.268		
Pneumonia		9(45.0)		18(43.9)		0.935		
Neonatal septicemia		3(15.0)		2(4.9)		0.176		
PDA		15(75.0)		18(43.9)		**0.022**		
Intracranial hemorrhage		6(30.0)		9(22.0)		0.493		

### Ang-1 and sCD105 expression levels in cord blood plasma

**Fig. 2:**
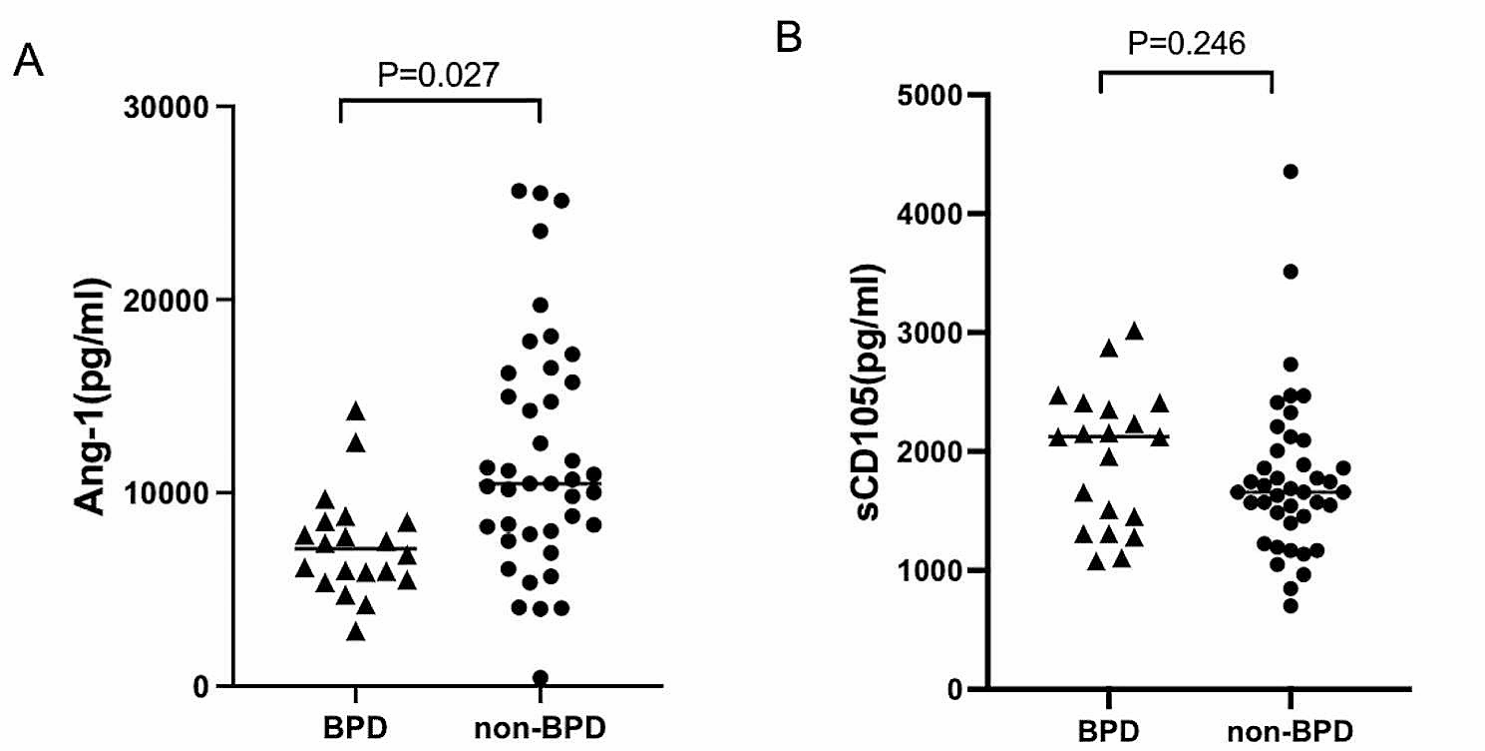
Comparison of Ang-1 and sCD105 concentrations in cord blood between the BPD and non-BPD groups

The cord blood Ang-1 and sCD105 expression levels in all samples were 8800.40 (6108.25–13445.04) pg/ml and 1715.94 (1426.09–2182.58) pg/ml, respectively. Moreover, the Ang-1 concentration level in the BPD group was lower than that in the non-BPD group (7105.43 (5617.01–8523.00) pg/ml vs. 10488.03 (7946.19–15962.77) pg/ml, respectively, *P* = 0.027). However, sCD105 concentration levels were not statistically different between the two groups (2121.74 (1346.37–2397.10) pg/ml vs. 1657.97 (1426.09–2049.28) pg/ml, respectively, *P* = 0.246) (Fig. 2).

### Analysis of risk factors associated with BPD in preterm infants

Binary logistic regression analysis was used to further explore Ang-1 levels as a predictor of the risk of BPD. According to the median Ang-1 concentration (8800.40 pg/ml), Ang-1 levels were categorized into the low level (≤ 8800.40 pg/ml) and high level (> 8800.40 pg/ml) groups. Table 2 shows the effect of low Ang-1 concentrations on the risk of developing BPD in preterm infants. After adjusting for gestational age and birth weight, the risk of BPD in preterm infants with Ang-1 concentrations of ≤ 8800.40 pg/ml was 10.720 times (95% CI: 1.793–64.096, *P* = 0.009) higher than that in preterm infants with Ang-1 concentrations of > 8800.40 pg/ml. Subsequent adjustments for antenatal steroid application did not completely weaken the association (OR, 8.577; 95% CI: 1.265–58.155, *P* = 0.028).

**Table 2:** Effect of low concentration levels of Ang-1 on the risk of developing BPD in preterm infants

	B	Wald χ^2^	*P*	OR	95%CI
*Model 1*	2.043	9.916	0.002	7.714	2.163–27.515
*Model 2*	2.372	6.760	0.009	10.720	1.793–64.096
*Model 3*	2.149	4.843	0.028	8.577	1.265–58.155

Ang−1 > 8800.40 pg/ml as reference

Model 1: Crude model

Model 2: Adjust for gestational age and birth weight

Model 3: Model 1 + adjust for antenatal steroids

## Discussion

BPD is a serious respiratory complication in preterm infants. There is an urgent clinical need for the early prognosis of the risk of developing BPD to avoid or minimize the occurrence of severe BPD. Currently, the diagnosis of BPD relies on clinical manifestations and oxygen demand; however, the pathophysiology of BPD in children changes after birth [16]. Current research suggests that the main pathological features of BPD include alveolar developmental delay and vascular dysfunction [17].

### Ang-1 and BPD

In this study, we found that the cord blood Ang-1 concentrations in children with BPD were significantly lower than those in children without BPD. Preterm infants with low Ang-1 concentrations have a much higher risk of developing BPD than those with high Ang-1 levels. Ang-1 promotes alveolar maturation and pulmonary vascularization, and affects the pathophysiological process of BPD, thereby reducing the incidence of BPD. Mohamed WA et al. reported a correlation between cord blood Ang-1 levels and the development of BPD. This article shows positive association of low Ang-1 in cord plasma and increased incidence of BPD in a 102 preterm infants sample [18]. Kim et al. showed that the peripheral blood level of Ang-1, a factor that promotes the formation of vascular endothelial growth factor, was lower in children with severe BPD than in those with non-BPD or mild BPD [19]. There have different aspects comparing to ours since (1) the peripheral blood specimens were collected on the first day of life, and (2) the grouping of preterm infants was different, as their study categorized them into severe BPD and non/mild BPD. The limitations of our study are that we only conducted a study of Ang-1 levels in umbilical cord blood of infants at this stage with BPD. Previous studies have evaluated the levels of potential markers associated with BPD at 36 weeks postmenstrual age [20]. This study found that BPD patients had lower levels of Ang-1 when compared to term infants with no lung pathology/BPD and BPD who developed pulmonary hypertension(PH). This is consistent with our findings. Ang-1 regulates alveolar development and pulmonary vascularization, and plays an important role in the development of BPD.

The lungs require an increased lung surface area for effective intrapulmonary gas exchange, which involves increasing the number of alveoli and blood vessels, as well as thinning the alveolar-capillary barrier. These changes primarily occur during the later stages of lung development [21]. In children with BPD, this critical late lung development is interrupted, leading to ineffective gas exchange and the therapeutic need for respiratory support in the form of positive pressure ventilation support and oxygen therapy [22, 23]. This suggests that abnormal early pulmonary vascular development plays a critical role in the pathogenesis of BPD. Ang-1 has anti-inflammatory, anti-apoptotic, anti-vascular leakage and vascular stabilisation functions on vascular endothelial cells, and has great potential for the repair and treatment of lung injury [24]. Ang-1 is an important mediator of angiogenesis. By establishing an experimental model of sepsis-induced BPD, Salimi U et al. found that Ang-1 kept endothelial cells quiescent, inhibited acute lung injury, and suppressed alveolar simplification [25]. During the formation of the pulmonary vascular system and normal development of the lungs, Ang-1 promotes the refinement of the spatially structured vascular network, which plays a pivotal role in the late plasticization of blood vessels.

### sCD105 and BPD

In this study, we compared the association of cord blood sCD105 concentration levels within BPD and non-BPD groups and found that cord blood sCD105 levels did not differ between the two groups. This finding is inconsistent with those of other studies. An increase in cord blood sCD105 levels is associated with the development of severe or moderate BPD in preterm infants born to pregnant mothers with preeclampsia [26]. There may be various reasons for the inconsistent results of this study. (1) The severity of BPD was not graded in this study, which may have affected the experimental results. (2) It may be due to disturbances in the internal environment of the pregnant mother, inflammatory damage, intrauterine hypoxia, or vascular endothelial damage due to preeclampsia, which all may have affected our findings.

Angiogenesis is necessary for alveolar formation during normal lung development, and disruption of angiogenesis during critical periods of lung growth may lead to lung hypoplasia [27]. Transforming growth factor-β(TGF-β) is involved in the regulation of angiogenesis and neovascularization [28]. Several studies have suggested that sCD105 may be a key molecule in determining the TGF-βsignaling pathway [29]. Moreover, sCD105 plays a regulatory role mainly in the TGF-β-ALK5-Smad2/3 signaling pathway. Elevated levels of Smad2/3 phosphorylation reduce angiogenesis and impair alveolarization [30]. The unbalanced high expression of sCD105 suggests that this process may underlie the disruption of developmental alveolization due to impaired pulmonary angiogenesis, which affects BPD development [30]. In the future, we need to further investigate the regulatory mechanism of sCD105 and its role in the development of BPD.

### Limitations

Although several angiogenic factors have been studied in lung development, these biomarkers are still in the early stages of exploratory discovery and their accuracy and reliability are suboptimal and require further clinical validation. Our study has some limitations. First, the sample size of this study was small and was taken only from Taizhou Hospital, therefore, this is a limited study and not representative of the entire population. Further multicenter, large-sample studies are required in the future. Second, the number of very preterm and ultralow birthweight infants in our study population was low. Third, the proportion of infants developing BPD was high in our study. There were some infants for whom we did not collect cord blood as well as did not obtain informed consent from the guardian. This part of missing data may affect the percentage of BPD. In addition, antenatal steroids therapy may influence the development of BPD. The percentage of antenatal steroids use was low in our study [18]. Finally, the development of BPD is associated with various factors during the postnatal period, including disease severity at birth, timeliness of resuscitation, postnatal complications, and therapeutic measures. We are continuing to collect peripheral blood samples from preterm infants at 24 and 72 h, and 7, 14, and 21 days after birth. In the near future, we will further explore the expression level of Ang-1 at different postnatal time points to better illustrate its correlation with BPD development. This is an important area and direction to look into in the pathogenesis of BPD.

## Conclusion

Our study indicated that Ang-1 levels in the cord blood of preterm infants may be associated the risk of BPD. There was no association between the sCD105 concentration and BPD in preterm infants. Next, we will investigate the mechanisms of Ang-1 and sCD105 in the development of BPD in preterm infants using large clinical samples. This will help in the early clinical diagnosis and prevention of BPD, and may provide a new therapeutic target.

## Data Availability

The raw data supporting the conclusions of this article will be made available by the authors, without undue reservation. [Contact: Jingyun Yang, email: 1592954680@qq.com]

## Abbreviations

BPD: Bronchopulmonary dysplasia
Ang-1: Angiopoietin-1
sCD105: S-endoglin
OR: Odds ratios
CI: Confidence interval
